# Practical implications of androgen receptor inhibitors for prostate cancer treatment

**DOI:** 10.37349/etat.2024.00234

**Published:** 2024-05-28

**Authors:** Fabio Campodonico, Luca Foppiani, Vittoria Campodonico, Carlo Introini

**Affiliations:** National Research Council (CNR), Italy; ^1^Department of Abdominal Surgery, Urology Unit, Galliera Hospital, 16128 Genova, Italy; ^2^Internal Medicine, Galliera Hospital, 16128 Genova, Italy; ^3^School of Pharmaceutical Sciences, University of Genova, 16132 Genova, Italy

**Keywords:** Androgen receptor inhibitors, antiandrogens, androgen deprivation therapy, prostate cancer, enzalutamide, apalutamide, darolutamide

## Abstract

Antiandrogens have been used for the treatment of prostate cancer as a single agent or in combination with hormone deprivation therapy. New generation antiandrogens act like androgen receptor inhibitors (ARIs). Their binding complex blocks the pathways of cellular proliferation and differentiation of the prostate. Enzalutamide, apalutamide and darolutamide are the new ARIs that demonstrated acceptable tolerability and toxicity, both active in hormone-sensitive and castration-resistant prostate cancer (CRPC). There is no evidence of superiority of one drug over the other, therefore the therapeutic choice depends on the safety profile in relation to the individual patient, their comorbidities and clinical condition. ARIs have also shown promising results in association with new drugs that are active on patients with metastatic CRPC carrying the mutated breast cancer gene (*BRCA*). Before undergoing new antiandrogenic therapies, patients should be evaluated for cardiological and metabolic risk and possible drug interactions.

Prostate cancer (PC) is the most prevalent malignancy in men although not the first in terms of cancer-specific mortality [1]. Androgen deprivation therapy (ADT) has been the cornerstone of systemic treatment used since the 1940s to improve symptoms and prolong survival in PC [2]. In the lasts two decades, anti-androgens such as flutamide, nilutamide and bicalutamide were used in combination with ADT to prevent tumor flare due to testosterone surge. In the therapeutic setting of castration-resistant PC (CRPC), attempts have been made to develop treatments for it. However, the treatments have shown modest efficacy, with a short response on PSA control and without significant impact in delaying the disease progression [3]. Among hormonal drugs abiraterone inhibits androgenic steroid synthesis by blocking the conversion of pregnenolone-like steroids into androgens, resulting in castration at the adrenal, testicular and tumor cell level [4]. The new generation of androgen receptor inhibitors (ARIs) has shown efficacy and good tolerability in the treatment of different stages of PC, contributing to improvements in overall survival in patients with various disease (Table 1). Enzalutamide, apalutamide and darolutamide are the new ARIs used for treating advanced PC. Ongoing clinical trials are investigating the drugs in various sequences and associations with other treatments. Recently the association of enzalutamide with Poly-ADP-Ribose-Polymerase (PARP) inhibitors in metastatic PC patients with breast cancer gene (*BRCA*) gene mutation has shown a promising result in reducing the disease progression and death [5]. Androgens regulate the function of normal prostate cells by activating androgen receptors (ARs) (Figure 1). These form a complex which is subsequently translocated from the cytoplasm to the nucleus where various pathways are activated, including those of cell proliferation, differentiation and prevention of apoptosis [6]. ARIs directly bind the AR blocking all downstream signaling events. First-generation antiandrogens did not completely block AR because they possess agonist activity on cells that express high levels of ARs (Figure 2). Differently, second generation ARIs have an antagonistic effect on AR [7].

**Table 1:** Main trials including ARIs for PC treatment

**Drug**	**Patient stage**	**Study**	**Benefit**
Enzalutamide	mCRPCa post-CT	AFFIRM 2012	+ OS
Enzalutamide	mCRPCa before-CT	PREVAIL 2014	+ PFS
Enzalutamide	mHSPCa	ENZAMET 2019	+ OS
Enzalutamide	mHSPCa	ARCHES 2019	+ OS
Enzalutamide	nmCRPCa, PSADT ≤ 10 ms	PROSPER 2018	+ OS
Enzalutamide	nmHSPCa, PSADT ≤ 9 ms	EMBARK 2020	+ MFS
Apalutamide	nmCRPCa, PSADT ≤ 10 ms	SPARTAN 2018	+ MFS
Apalutamide	mHSPCa	TITAN 2019	+ OS
Darolutamide	nmCRPCa, PSADT ≤ 10 ms	ARAMIS 2019	+ OS
Darolutamide	mHSPC	ARASENS 2022	+ OS

CT: computed tomography; +: increasing benefit; mHSPC: metastatic hormone sensitive prostate cancer

**Figure 1:**
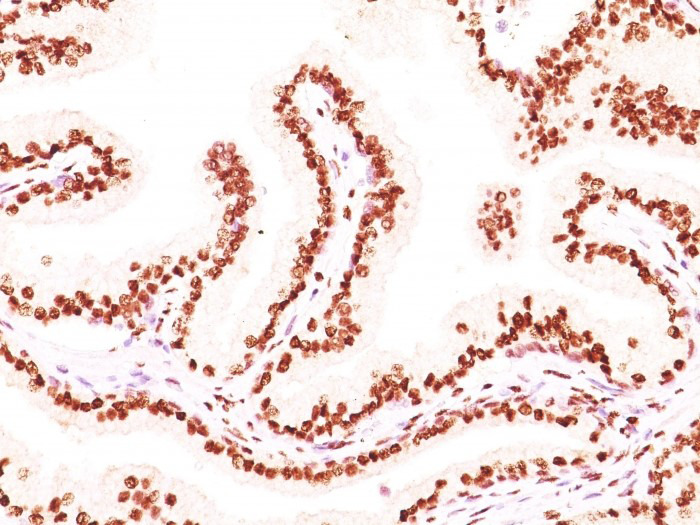
Formalin-fixed, paraffin-embedded human prostate carcinoma stained with AR monoclonal antibody (image source: AR441 + DHTR/882, NeoBiotechnologies Inc. Union City, CA, USA). AR: androgen receptor

**Figure 2:**
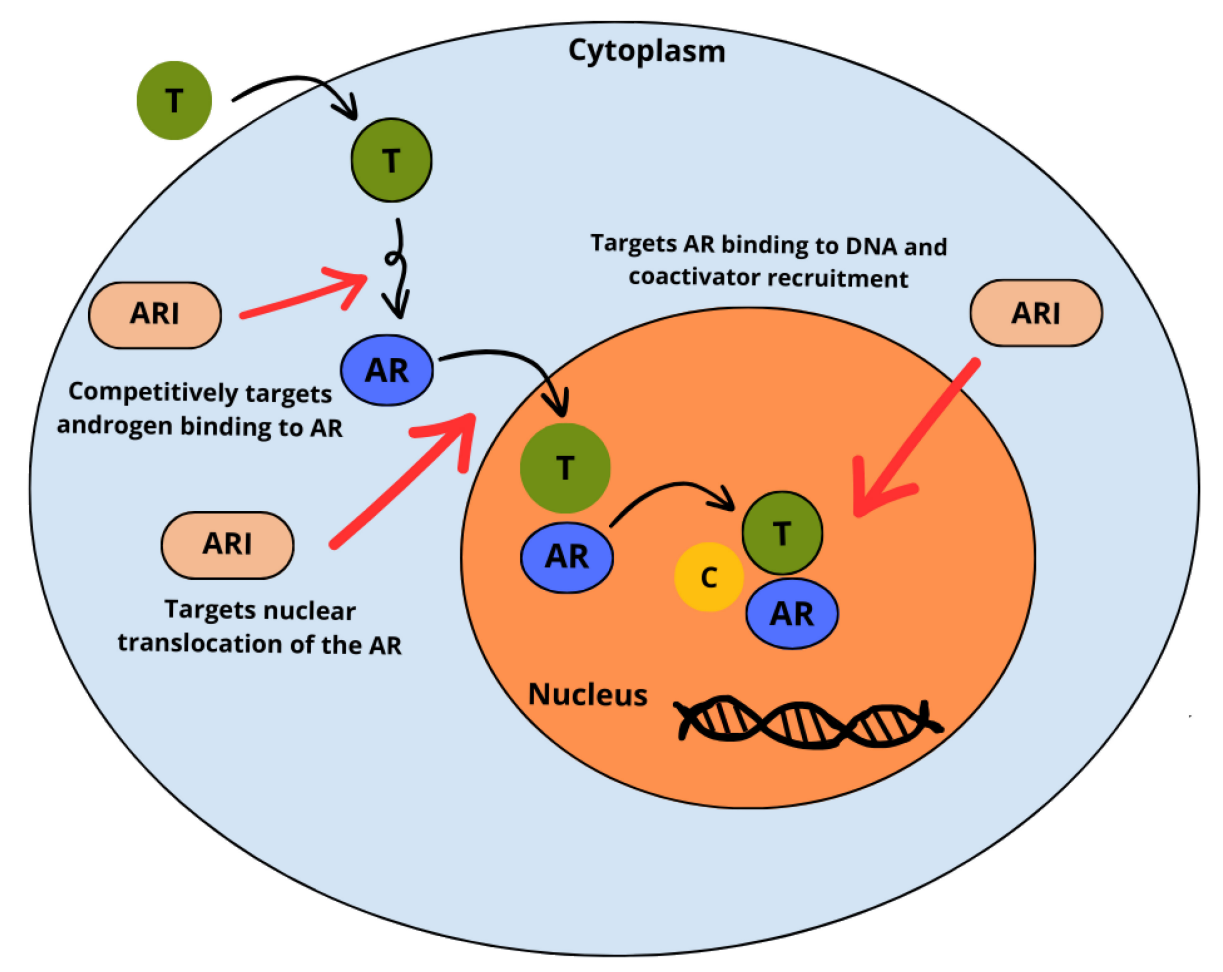
Multi-step inhibitory activity of ARIs against T at the AR level, intra-cytoplasmic AR translocation, and DNA-AR complex binding. AR: androgen receptor; ARI: AR inhibitor; T: testosterone; C: coactivator *Note.* Adapted from “Enzalutamide for the treatment of metastatic castration-resistant prostate cancer,” by Rodriguez-Vida A, Galazi M, Rudman S, Chowdhury S, Sternberg CN. Drug Des Devel Ther. 2015;9:3325–39 (https://doi.org/10.2147/DDDT.S69433). CC BY-NC.

Enzalutamide, the first second**-**generation antiandrogen, is chemically related to bicalutamide but it has five- to eight-fold higher binding affinity for AR than bicalutamide. Enzalutamide was approved after the AFFIRM study. The study compared two groups of patients having metastatic CRPC (mCRPC) and pre-treated with chemotherapy. The group treated with enzalutamide had greater overall survival (18 months *versus* 13 months), PSA response increase and delayed time to PSA progression [8]. In pre-chemo treated patients with mCRPC (PREVAIL trial), enzalutamide also showed an increase in progression-free survival [9]. In metastatic hormone sensitive PC (mHSPC), enzalutamide plus ADT, including 20% of patients receiving local therapy or docetaxel, demonstrated efficacy in overall survival, better PSA increase interval and longer time to clinical progression (ARCHES trial) [10, 11]. Better outcomes and overall survival were also reported in the ENZAMET trial comparing enzalutamide with older AR antagonists; both arms associated with ADT with or without docetaxel [12]. Enzalutamide was also tested in patients with non-metastatic CRPC, based on negative computed tomography (CT) scan and bone scan, PSA ≥ 2 ng/mL, and PSA doubling time ≤ 10 months. The study (PROSPER trial) achieved the primary endpoint of improved metastasis-free survival from 14 months to 36 months, delayed PSA progression as well as increased time to start subsequent treatments [13]. Enzalutamide resulted in higher overall survival in nmCRPC compared to placebo treatment, 67 months *versus* 56 months, respectively [14]. Enzalutamide has also been studied in patients with high-risk biochemical relapse after local treatment (EMBARK trial), with PSA ≥ 2 ng/mL above the nadir after radiotherapy, and PSA ≥ 1 ng/mL after radical prostatectomy, with or without postoperative radiotherapy, and associated with a PSA doubling-time ≤ 9 months [15].

Apalutamide is another oral, nonsteroidal ARI that can be prescribed in patients with nmCRPC and mHSPC. In the SPARTAN trial, patients with nmCRPC and PSA doubling-time < 10 months, treated with apalutamide, demonstrated a metastasis-free survival of 40 months compared to 16 months of the placebo arm [16]. In the mHSPC setting (TITAN trial), the overall survival improved with apalutamide compared to placebo [17]. A small group of patients received also prior local therapy and docetaxel, 16% and 11%, respectively.

Darolutamide is a new ARI that resists numerous AR mutations. It has minimal penetration through the blood-brain barrier, hence adverse events of the central nervous system are reduced [18]. The benefit of this drug was observed in terms of overall survival and reduction of side effects (ARAMIS trial). Darolutamide has also recently been investigated in the mHSPC setting (ARASENS trial), demonstrating excellent overall survival compared to placebo [19].

The development of resistance to androgen pathway—targeted agents is crucial to PC lethality. Pre-clinical studies indicated that drug-resistant condition can be attenuated or reversed by epigenetic modulation. This hypothesis in CRPC has been tested in a phase I/II trial of the histone deacetylase inhibitor panobinostat in combination with bicalutamide in patients who progressed on bicalutamide and/or other first-generation antiandrogen. The results provide evidence that compared to historic controls treated with bicalutamide alone, the combination of bicalutamide with high-dose panobinostat administered intermittently delayed radiographic progression of the disease. An initial proof-of-concept support for the potential effectiveness of epigenetic therapy to reverse or delay antiandrogen-resistant CRPC has been tested, and should be confirmed with currently available, more potent antiandrogens [20].

Recently, the combination of new ARIs with an oral PARP inhibitor (PARPi) has been evaluated in mCRPC. PARPi associated with novel hormonal agents ARIs demonstrated to be effective as first-line therapy in patients with *HRR* gene mutations. Based on trial TALAPRO-2 enzalutamide associated with talazoparib, in patients with *BRCA*-mutated mCRPC, the primary end-point of radiographic progression-free survival improved compared to placebo. Common side effects were anemia, fatigue, asthenia and nausea [5]. In the CASPER trial the association between rucaparib and enzalutamide was tested, which actually is under data evaluation [21].

Data from clinical trials have shown several adverse events with enzalutamide, such as asthenia or fatigue, muscle and bone pain, hot flashes, hypertension, falls and headache, paresthesia, cognitive impairment, seizures, major cardiovascular events, hypothyroidism. Finally, posterior reversible encephalopathy syndrome has been rarely reported [22]. Therapy with enzalutamide may be associated with QT corrected (QTc) prolongation, which increases the risk of arrhythmias, such as torsade de pointes and ventricular tachycardia. It is well ascertained that testosterone regulates ventricular repolarization by the upregulation of K^+^ currents and the suppression of Ca^2+^ currents, leading to a shortening effect on QT. Hence, drugs which impair testosterone action may affect QT interval. Caution is urged with enzalutamide in patients with a history of seizures, brain injury, stroke and brain tumors, alcoholism or concomitant use of drugs that reduce the seizure threshold. From a pathophysiological point of view, seizures are related to the capacity of enzalutamide to cross the blood-brain barrier and to antagonize the gamma aminobutyric acid-alfa receptor in the brain. However, in clinical practice the rate of seizures is low (< 1%). In patients undergoing treatment with direct oral anticoagulants (DOAC), physicians must take into account the interactions with enzalutamide, which is a potent cytochrome P450 (CYP) 3A4 enzyme inducer. CYP3A4 is responsible for apixaban or rivaroxaban metabolism. Enzalutamide should not therefore be coadministered with these two DOAC for the risk of reducing their plasmatic concentrations, and hence their anticoagulant activity [23].

Apalutamide has seven- to ten-fold greater affinity for AR than bicalutamide. The most common adverse events linked to apalutamide in clinical trials were fatigue, rash, hypertension, falls, fracture, and less frequently hypothyroidism. Rash, which is the most common grade ≥ 3 adverse event, is macular or maculopapular, occurring about 80 days after starting therapy. Topical hydrocortisone cream can be applied for localized symptoms. If symptoms become more severe patients need to discontinue the use of the drug and oral steroid and eventually oral antihistamines are needed [24]. Apalutamide was shown to have a relationship with a concentration-dependent increase in the QTc interval; accordingly, electrocardiogram and electrolyte (calcium, potassium, magnesium) assessment should be performed before and during therapy. Fracture risk increases with apalutamide and dual energy X-ray absorptiometry should be performed at baseline and yearly after the start of therapy. Supplementation of colecalciferol is advised and bone-building drugs may be required [25]. Apalutamide may induce UDP-glucuronosyl transferase (UGT). Levothyroxine and thyroxine are substrates of UGT; hence, patients on therapy with apalutamide who are receiving levothyroxine should be checked for the need to increase daily dose. TSH levels increase about 4 months after treatment initiation, and FT3 and FT4 should be monitored regularly both in patients already on replacement therapy and in those who are not. Apalutamide is metabolized by CYP2C8 and CYP3A4. A range of inhibitors and substrates of CYP3A4, CYP2C19 and P-glycoprotein are assumed to interact with apalutamide resulting in loss of activity. Concomitant use of inhibitors of CYP2C8 such as gemfibrozil, clarithromycin and inhibitors of CYP3A4, such as itraconazole and fluconazole, may increase drug exposure. By contrast, inducers of CYP3A4 such as carbamazepine, phenobarbital and rifampicin can decrease plasmatic drug concentration [23]. In turn, apalutamide can decrease exposure to several drugs such as omeprazole, warfarin, midazolam, rosuvastatin, clopidogrel, quetiapine. Common alteration in laboratory examinations include: leukopenia, anemia, hyperglycemia, hypercolesterolemia, hypertriglyceridemia. The possible worsening of metabolic profile induced by apalutamide requires prompt modifications in both lifestyles and initiation of specific pharmacological therapy.

Darolutamide possesses a similar mechanism of action to enzalutamide and apalutamide; it has an eight-fold greater affinity for the AR compared to enzalutamide. By comparing the adverse effect profile described in the different registration trials, darolutamide was found to have a more favourable safety profile without impairing quality of life. The most frequent adverse effect observed in clinical trials was fatigue and asthenia. However, fatigue was reported in a lower percentage of patients (15.8%) in the ARAMIS trial than in PROSPER (enzalutamide) and SPARTAN (apalutamide) trials [26]. No increased incidence of seizures, falls, fracture, cognitive disorder compared to placebo was observed. However, heart failure, ischemic heart disease and hypertension have been associated with this drug [27]. Dosage was reduced due to adverse reactions that occurred in a few patients (6%) receiving darolutamide, most frequently because of fatigue and hypertension. This drug has a chemical structure which significantly reduces its penetration into the blood-brain barrier. Accordingly, the risk of seizure, mental impairment and falls decreased campared to enzalutamide and apalutamide [28]. Laboratory test abnormalities occurring in patients being treated with therapy with darolutamide were leukopenia, increased aspartate aminotransferase levels, and increased bilirubin levels. Darolutamide shows a low incidence of interactions with commonly prescribed medications.

## General considerations with the use of second-generation ARIs

Since patients with uncontrolled hypertension or recent cardiac or cerebral ischemic events were excluded from the studies assessing the efficacy of the ARIs, it is not possible to assess properly the impact of these drugs on the cardiovascular system. However, clinical trials have shown a clear increased risk of hypertension particularly with the use of enzalutamide (risk of all-grade and high-grade hypertension increased by 26.2%), and in lesser amount, of cardiovascular events such as myocardial infarction. Based on these findings, cardiovascular risk stratification (low, moderate, and high) should be assessed before starting therapy and the various cardiovascular risk factors (hypertension, diabetes mellitus, and dyslipidemia) should be optimized before, during, and after therapy in patients with PC receiving ADT [20]. Patients with nmCRPC in whom second-generation ARIs may be indicated are generally old (median age 75 years), frail, and with several cardiovascular comorbidities (hyperlipidemia, hypertension in more that two-thirds of patients, arrhythmias and congestive heart failure) and comedications (antithrombotic agents, lipid-modifying agents, beta-blockers, antimicrobial agents). The drug-drug interaction represents a major clinical concern in these patients. Among second-generation ARIs, only darolutamide pharmacokinetics remained unmodified despite the use of co-medications.

## Suggestions for the use of second-generation ARIs in clinical practice

In patients with uncontrolled cardiovascular disease or many cardiovascular risk factors, enzalutamide or apalutamide should be avoided due to the increased risk of associated hypertension. Darolutamide has the lowest risk of hypertension. Darolutamide shows a lower risk of central nervous-related events compared with apalutamide and enzalutamide, with a significantly reduced absolute risk for mental impairment and hence for falls and fractures. Apalutamide shows the most significant drug-drug interactions, with possible harmful consequences in patients taking multiple medications. Currently, there are no data showing one of the new ARIs having more efficacy than the others, in the different settings of PC. The treatment choice should be balanced considering the drug safety profile and the single patient clinical condition, including comorbidities and associated polypharmacotherapies.

## Abbreviations

ADT: androgen deprivation therapy
ARIs: androgen receptor inhibitors
ARs: androgen receptors
CRPC: castration-resistant prostate cancer
CT: computed tomography
CYP: cytochrome P450
mCRPC: metastatic castration-resistant prostate cancer
mHSPC: metastatic hormone sensitive prostate cancer
PC: prostate cancer

## Declarations

### Copyright

© The Author(s) 2024.
